# Supplementary material for: The Construction and Exploration of a Comprehensive MicroRNA Centered Regulatory Network in Foxtail Millet (Setaria italica L.)
Source: Front Plant Sci. 2022 May 6;13:848474. doi: 10.3389/fpls.2022.848474 (PMC9121102; doi:10.3389/fpls.2022.848474)
Supplement: Supplementary file 6 [file Data_Sheet_6.zip › Sit-miRN3020_Seita.3G267200.1_130_TPlot.pdf]

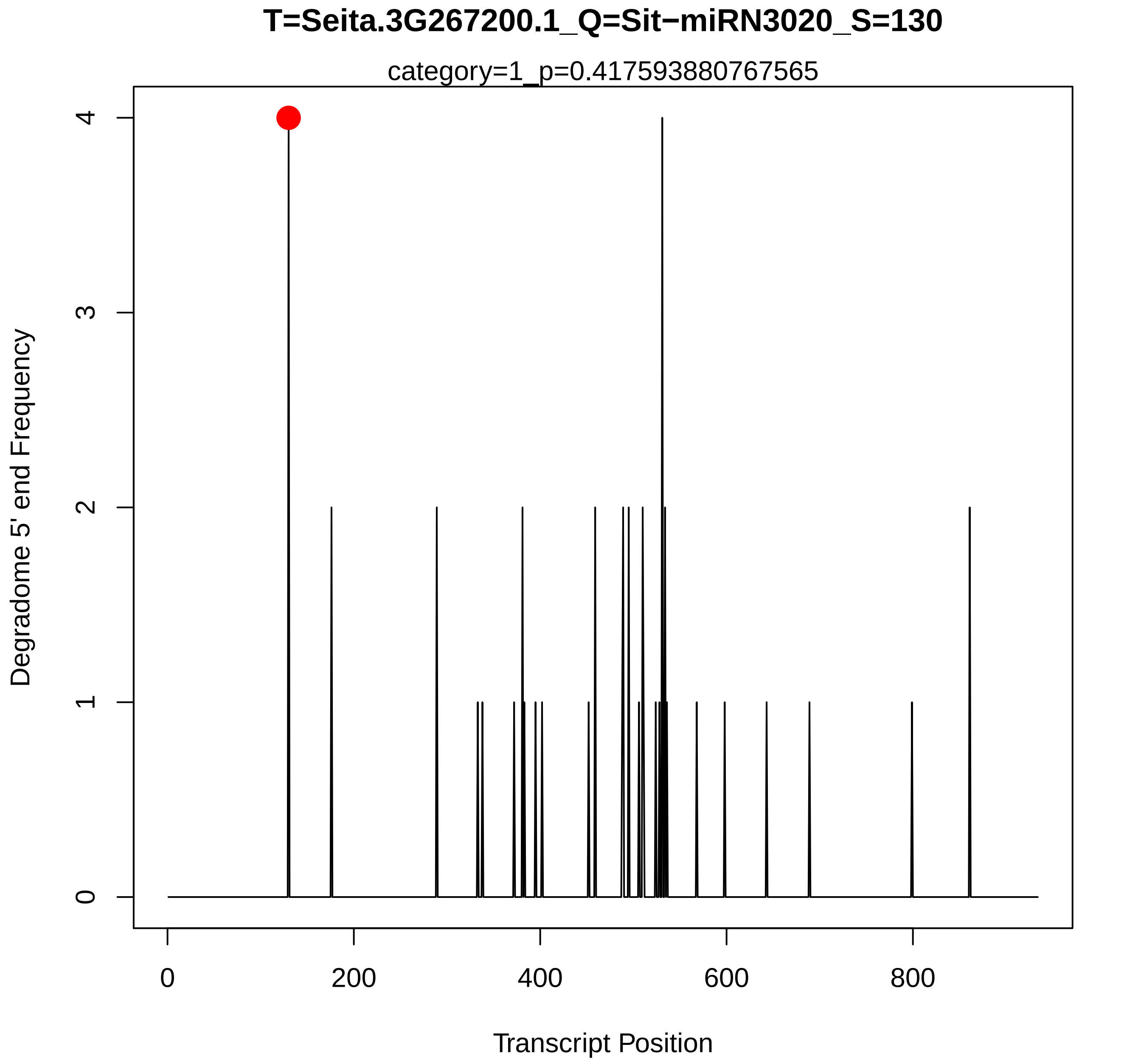
T=Seita.3G267200.1_Q=Sit−miRN3020_S=130
category=1_p=0.417593880767565
Degradome 5' end Frequency
0
1
2
3
4
0
200
400
600
800
Transcript Position
